# Small molecule inhibitor screening identifified HSP90 inhibitor 17-AAG as potential therapeutic agent for gallbladder cancer

**DOI:** 10.18632/oncotarget.15410

**Published:** 2017-02-16

**Authors:** Helga Weber, José R. Valbuena, Mustafa A. Barbhuiya, Stefan Stein, Hana Kunkel, Patricia García, Carolina Bizama, Ismael Riquelme, Jaime A. Espinoza, Stephen E. Kurtz, Jeffrey W. Tyner, Juan Francisco Calderon, Alejandro H. Corvalán, Manuel Grez, Akhilesh Pandey, Pamela Leal-Rojas, Juan C. Roa

**Affiliations:** ^1^ Center of Excellence in Traslational Medicine (CEMT) and Scientific and Technological Bioresource Nucleus (BIOREN), Universidad de La Frontera, Temuco, Chile; ^2^ Department of Pathology, Faculty of Medicine, Temuco, Chile; ^3^ Department of Pathology, School of Medicine, Pontificia Universidad Católica de Chile, Santiago, Chile; ^4^ McKusick-Nathans Institute of Genetic Medicine, The Johns Hopkins University School of Medicine, Baltimore, MD, USA; ^5^ Gene Therapy Unit, Institute for Tumor Biology and Experimental Therapy, Georg-Speyer-Haus, Frankfurt, Germany; ^6^ Center for Investigation in Translational Oncology (CITO), Advanced Center for Chronic Diseases (ACCDiS), Millennium Institute on Immunology and Immunotherapy, School of Medicine, Pontificia Universidad Católica de Chile, Santiago, Chile; ^7^ SciLifeLab, Division of Genome Biology, Department of Medical Biochemistry and Biophysics, Karolinska Institutet, Solna, Stockholm, Sweden; ^8^ Division of Hematology and Medical Oncology, Cell, Developmental and Cancer Biology, Oregon Health and Science University, Portland, OR, USA; ^9^ Center for Genetics and Genomics, School of Medicine, Clínica Alemana de Santiago - Universidad del Desarrollo, Santiago, Chile; ^10^ Department of Hematology Oncology, School of Medicine, Pontificia Universidad Católica de Chile, Santiago, Chile; ^11^ Department of Biological Chemistry, Department of Oncology, Department of Pathology, The Johns Hopkins University School of Medicine Baltimore, MD, USA

**Keywords:** gallbladder cancer, HSP90 inhibitors, geldanamycin, 17-AAG, gallbladder cancer xenografts

## Abstract

Gallbladder cancer (GBC) is a lethal cancer with poor prognosis associated with high invasiveness and poor response to chemotherapy and radiotherapy. New therapeutic approaches are urgently needed in order to improve survival and response rates of GBC patients. We screened 130 small molecule inhibitors on a panel of seven GBC cell lines and identified the HSP90 inhibitor 17-AAG as one of the most potent inhibitory drugs across the different lines. We tested the antitumor efficacy of 17-AAG and geldanamycin (GA) *in vitro* and in a subcutaneous preclinical tumor model NOD-SCID mice. We also evaluated the expression of HSP90 by immunohistochemistry in human GBC tumors.

*In vitro* assays showed that 17-AAG and GA significantly reduced the expression of HSP90 target proteins, including EGFR, AKT, phospho-AKT, Cyclin B1, phospho-ERK and Cyclin D1. These molecular changes were consistent with reduced cell viability and cell migration and promotion of G2/M cell cycle arrest and apoptosis observed in our *in vitro* studies.

*In vivo*, 17-AAG showed efficacy in reducing subcutaneous tumors size, exhibiting a 69.6% reduction in tumor size in the treatment group compared to control mice (*p* < 0.05).

The HSP90 immunohistochemical staining was seen in 182/209 cases of GBC (87%) and it was strongly expressed in 70 cases (33%), moderately in 58 cases (28%), and weakly in 54 cases (26%).

Our pre-clinical observations strongly suggest that the inhibition of HSP90 function by HSP90 inhibitors is a promising therapeutic strategy for gallbladder cancer that may benefit from new HSP90 inhibitors currently in development.

## INTRODUCTION

Gallbladder cancer (GBC) is the most common type of biliary-tract carcinomas. It has a high morbidity and poor prognosis as it is often associated with early metastasis and invasiveness [1]. GBC tumors affect thousands of individuals worldwide (annual incidence of 2.2/100,000), but has a much higher impact and mortality in Chile, China, Japan and India [2]. Complete surgical resection is the only potentially curative approach in early stages of the disease. However, most patients cannot undergo surgery since GBC is usually diagnosed at much more advanced stages [3]. Chemotherapy has been the main treatment option in circumscribed, advanced or metastatic biliary-tract carcinoma. A combination of chemotherapy with gemcitabine and a platinum-based agent is regarded as the standard treatment for patients with advanced biliary-tract cancer. However, the response to standard chemotherapy and radiotherapy is extremely poor, with modest impact on overall survival [4]. Therefore, new therapeutic options are needed to improve survival and response rates. Understanding the signaling pathways in GBC, such as Hedgehog, PI3K/AKT/mTOR, Notch, ErbB, MAPK and angiogenesis, holds great promise for the development of new effective treatment strategies [2].

High-troughput, rapid small molecule inhibitor screenings to identify therapeutic targets for cancer become tractable with the development of new technologies [5]. This method has been used so far to screen compounds for treating a number of heamatological malignancies [6]. We employed the same method to screen 130 potential treatment drugs in gallbladder cancer cell lines and identified a set of small molecule inhibitors including 17AAG, Velcade, Volasartib and YM-155 as the most potent growth inhibitors of these cell lines. We subsequently tested the HSP90 inhibitor 17AAG in pre-clinical cancer models.

HSP90 is a ubiquitous ATP-dependent molecular chaperone that facilitates the maturation of more than 200 downstream proteins, including key mediators of signal transduction, cell-cycle control and transcriptional regulation [7]. HSP90 also stabilizes numerous oncoproteins that facilitate malignant progression and maintain transformed cellular phenotypes as well as enables tumor cells to escape apoptotic death [8]. Its deregulation has been associated with the pathogenesis of various human cancers [9–11]. The use of inhibitors against HSP90 has been proposed as an attractive therapeutic strategy in numerous tumors based on the fact that HSP90 clients comprise proteins that contribute to all of the six hallmarks of cancer Therefore, its inhibition could simultaneously interfere with key pathophysiological processes of tumor cells, leading to more effective clinical results [9].

Geldanamycin (GA), a benzoquinone ansamycin antibiotic isolated from *Streptomyces hygroscopicus*, is a first generation HSP90 inhibitor with potent anti-tumor effects *in vitro*. However, it was never evaluated in clinical trials mainly due to metabolic instability, limited solubility, and induction of severe hepatotoxicity in animals [12]. 17-AAG (Tanespimycin), an analog molecule chemically derived from GA, was the first HSP90 inhibitor to enter clinical trials [13]. In preclinicals models it has shown antitumor activity in various types of cancer, such as colon, breast, ovarian, and melanoma tumors [12, 14].

In this study we systematically screened 130 small molecule for gallbladder cancer inhibition. We evaluated antitumoral activity of HSP90 inhibitors, 17-AAG and geldanamycin, *in vitro* and on a preclinical subcutaneous tumor model and showed the potential of the 17-AAG for further clinical investigations.

## RESULTS

### Small molecule inhibitors with therapeutic potential for GBC

**Figure 1:**
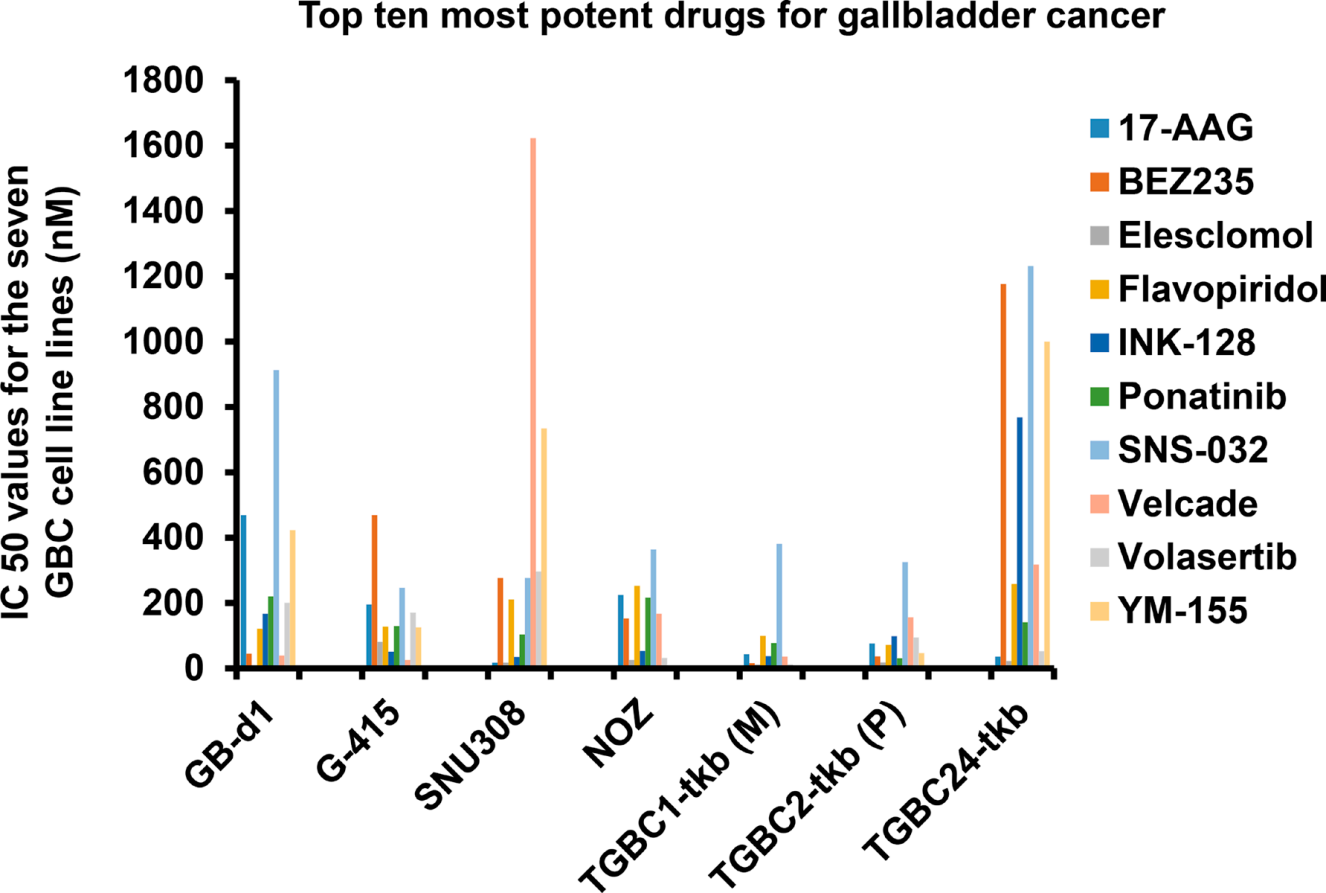
Top five most potent drugs for gallbladder cancer Seven human gallbladder cancer cell lines GB-d1, G415, SNU308, NOZ, TGBC1TKB, TGBC2TKB and TGBC24TKB were used for the rapid small molecule inhibitor screen including a panel of 130 small molecule inhibitors. Cell viability testing was carried out on the small-molecule inhibitor screening plates and synergy plates using a cell proliferation assay.

Based on previous publications by the co-authors of this study about the methodology high-throughput rapid small molecule inhibitor screening [5, 6], we pre-selected drugs from the FDA approved list of anti-cancer kinase and other small molecule inhibitors that were computationally and genetically (siRNA screening) tested in series of cancer cell lines. We adopted 130 drugs taking cue from those previous studies (Supplementary Table 1). In the rapid screen of these 130 drugs, we identified small molecules inhibitors including 17-AAG (Tanespimycin), Eleslomol, Velcade, Volasartib and YM-155 as the five most potent drugs across GBC cell lines. (Figure 1). Most of these drugs are either in clinical trials or have been determined to be effective against a wide range of cancers in preclinical tests. However, these molecules have not been investigated for their efficacy in GBC. It is important to note that all the seven GBC cell lines showed resistance against a series of widely used antitumoral drugs included in the screen. The IC_50_ values for the seven GBC cell lines of the drugs tested is provided in Figure 1 and Supplementary Table 1. These drugs are known to target many different kinases and receptors and have proved effective in other types of cancer. The results corroborate with the lack of effective chemotherapy-based treatment for GBC. Notably, five of them proved to be potent against the seven GBC cell lines investigated. Among these five candidates, we selected the HSP90 inhibitor 17-AAG (Tanespimycin) for pre-clinical validation as a potential therapeutic molecule for GBC.

### 17-AAG and GA reduce cell viability and cell migration in GBC cell lines *in vitro*

Due to HSP90 expression was highly upregulated in G-415 and GB-d1, compared with the other studied cell lines (data not shown), these cells lines were selected for further assays.

**Figure 2:**
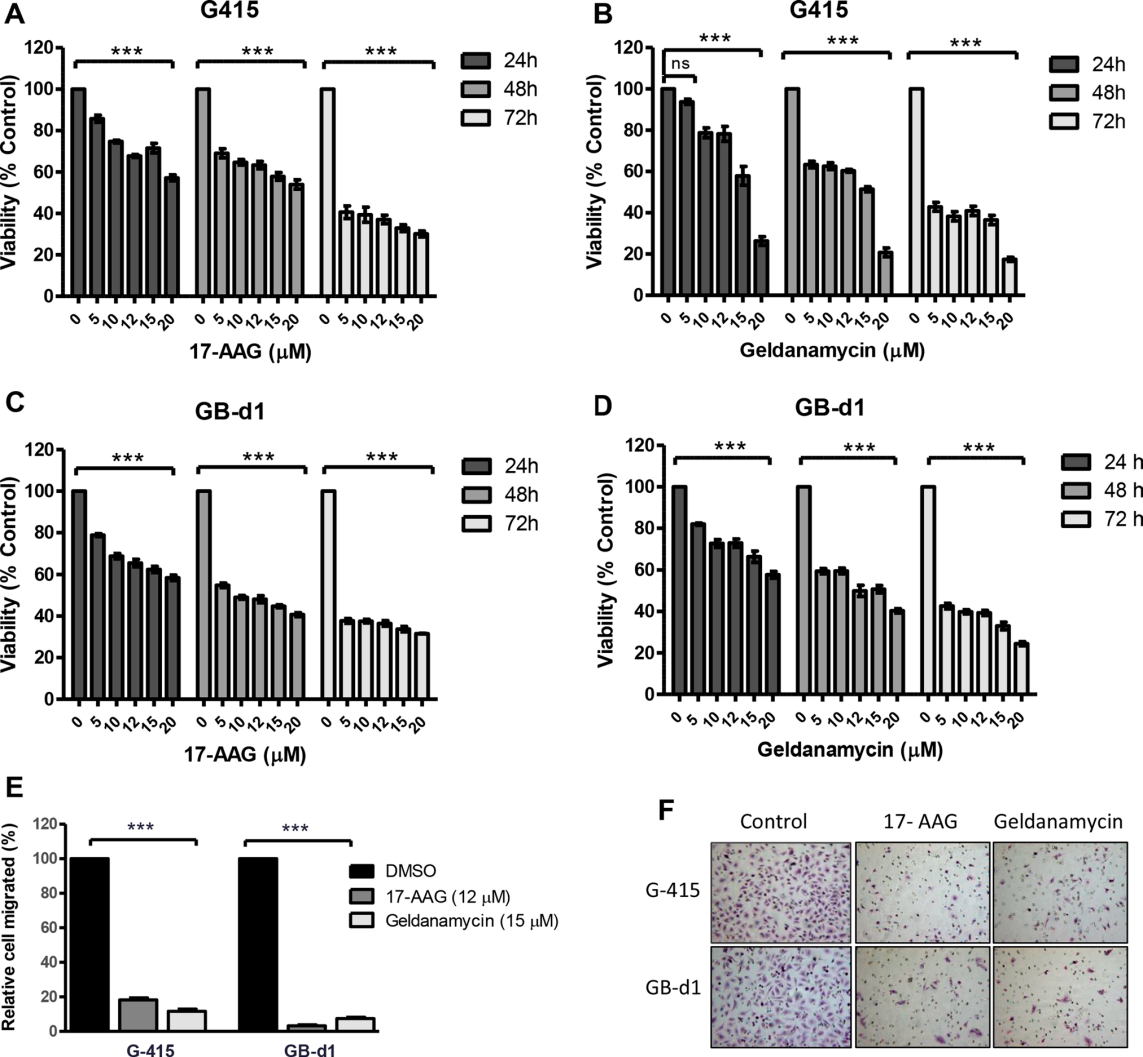
*In vitro* effects of 17-AAG and GA on cell growth and cell migration in two GBC cell lines (**A**, **B**) G-415 and (**C**, **D**) GB-d1 cells were treated with increasing concentrations of 17-AAG or GA. Cell viability was determined after 24, 48, and 72 hours of treatment. Data are shown as mean ± SD of at least three independent experiments in quintuplicate (***P < 0.001; ns: not significant). (**E**, **F**) Cell migration was evaluated in G-415 and GB-d1 cells treated with 17-AAG or GA (12 μM and 15 μM, respectively) for 24 hours. Control cells received an equivalent amount of solvent only. Data are shown as mean ± SD (***P < 0.001).

Cell viability was analyzed by MTS assay after treatment with increasing concentrations of GA or 17-AAG for 24, 48, and 72 hours. As shown in Figure 2A–2DA, 17-AAG and GA significantly reduced cell viability in both cell lines as early as 24 hours after exposure to the inhibitors (*P* < 0.001).

To establish the effect of 17-AAG and GA on cell migration, GBC cell lines were exposed to 17-AAG (12 μM), GA (15 μM), or 0.01% DMSOfor 24 hours. After this time, migration rates were significantly lower in treated versus untreated cells. Relative migration rates observed in G-415 were 18.3% (17-AAG) and 11.7% (GA) (*P* < 0.001) compared to the DMSO control, while GB-d1 showed 3.4% (17-AAG) and 7.4% (GA) (*P* < 0.001). (Figure 2E and 2F).

### Exposure to 17-AAG and GA reduces *in vitro* expression of HSP90 target proteins in GBC cells *in vitro*

**Figure 3:**
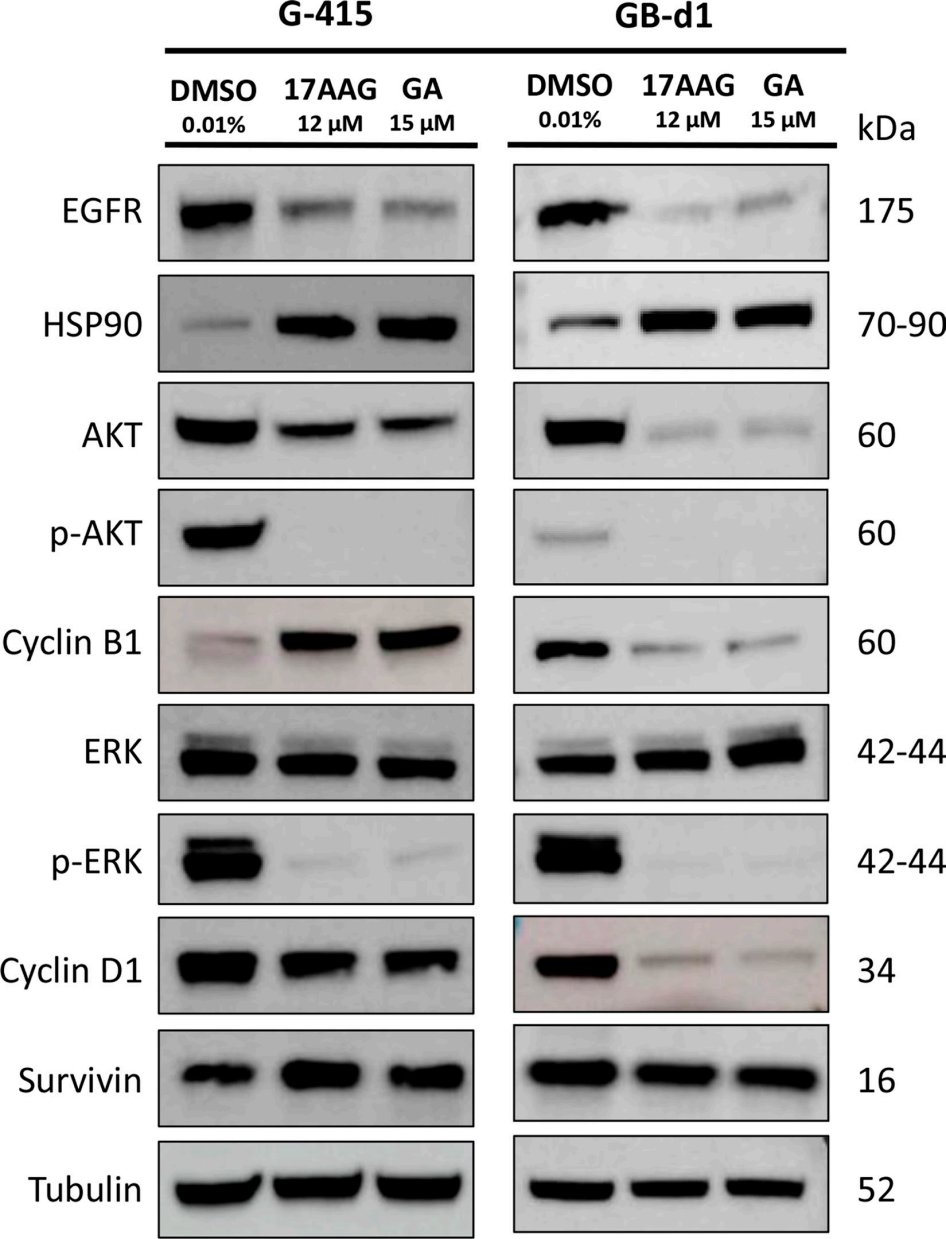
*In vitro* effects of 17-AAG and GA on HSP90 expression and on HSP90 client protein expression in two GBC cell lines G-415 and GB-d1 cells were treated with 17-AAG or GA (12 μM and 15 μM, respectively) for 24 hours. Control cells received an equivalent amount of solvent only. Western blot analysis was carried out using antibodies against HSP90 and HSP90 clients proteins. Protein loading was normalized using an antibody recognizing α-tubulin.

To further investigate the *in vitro* effects of 17-AAG and GA in GBC cell lines, we evaluated the expression of HSP90 and target proteins by immunoblotting. Cells were exposed to 17-AAG (12 μM), GA (15 μM) or DMSO for 24 hours and were lysed and analyzed by western blot using commercial antibodies. As shown in Figure 3, increased levels of HSP90 were observed upon HSP90 inhibition in G-415 and GB-d1 cells lines. On the other hand, HSP90 target proteins, EGFR, AKT, phospho-AKT, phospho-ERK and Cyclin D1 were strongly inhibited by 17-AAG or GA treatments in both cell lines. Treatment with either HSP90 inhibitors markedly decreased Cyclin B1 expression in Gb-d1 cells, but enhanced its expression in G-415 cells. No significant changes were observed in total ERK or survivin protein expression under the treatment conditions assayed.

### 17-AAG and GA treatment lead to G2/M cell cycle arrest and induces apotosis in GBC cells *in vitro*

**Figure 4:**
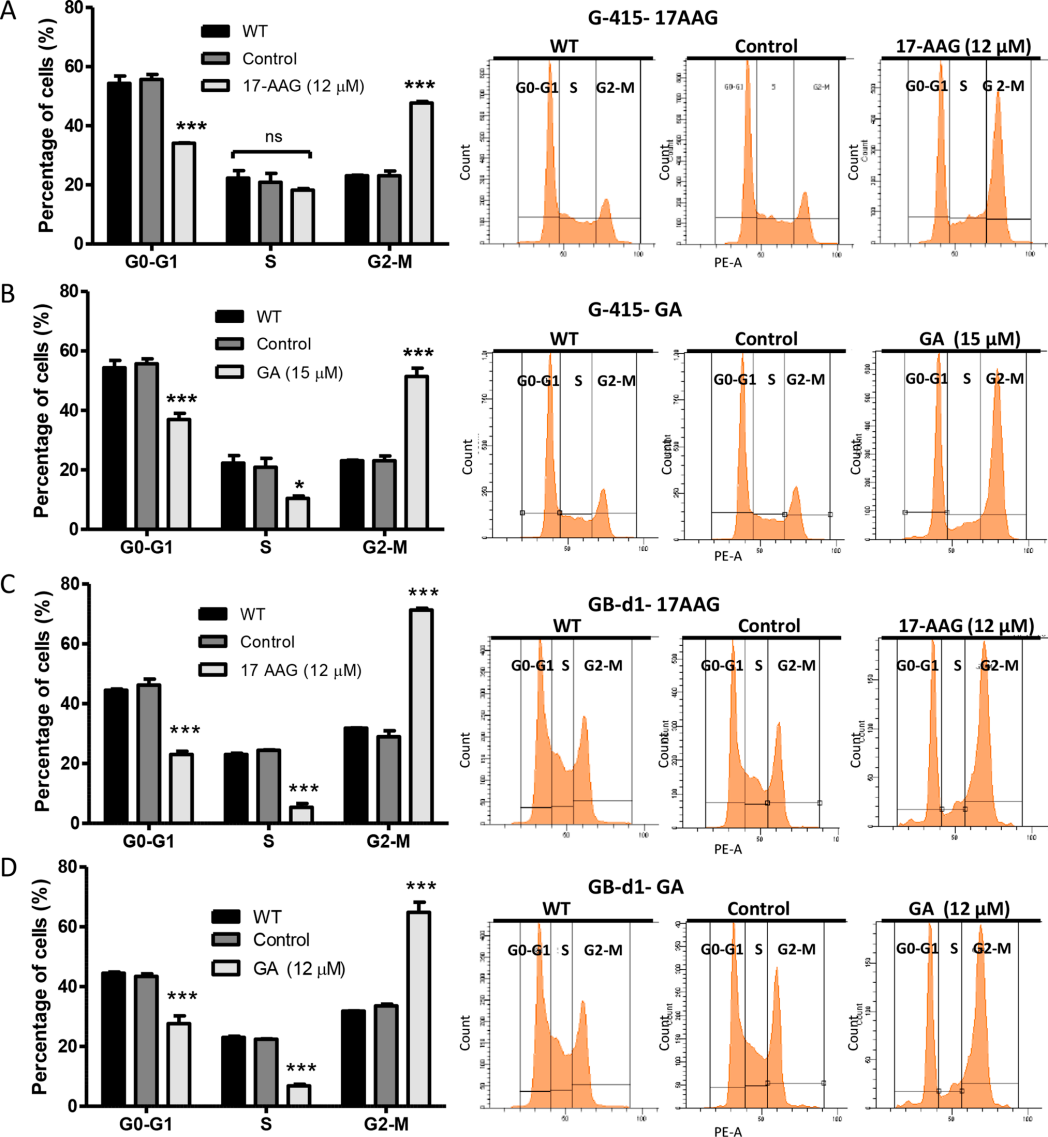
Effect of 17-AAG and GA on cell cycle on human GBC cells (**A** and **B**) G-415 and (**C** and **D**) GB-d1 cells were treated with dimethylsulfoxide or 12 μM and 20 μM of 17-AAG or with 15 μM and 20 μM of GA for 24 h. Cells were processed for analysis of cell cycle distribution by flow cytometry. Data are shown as mean ± SD of three independent samples and are representative of two independent experiments. (***P < 0.001; ns: not significant).

To determine whether the antiproliferative effect of 17-AAG and GA was due to cell cycle arrest, G-415 and GB-d1 cells were treated either with 17-AAG (12 μM), GA (15 μM), or DMSO for 24 hours. Cell cycle fractions were determined by PI-DNA staining and FACS analysis. After 24 hours of exposure, 17-AAG and GA treatments resulted in a statistically significant increase of G2/M phase cells compared with the controls, both in G-415 and in GB-d1 cells (*P* < 0.001). These results were accompanied by a decrease in G0/G1 and S phase populations. (Figure 4).

**Figure 5:**
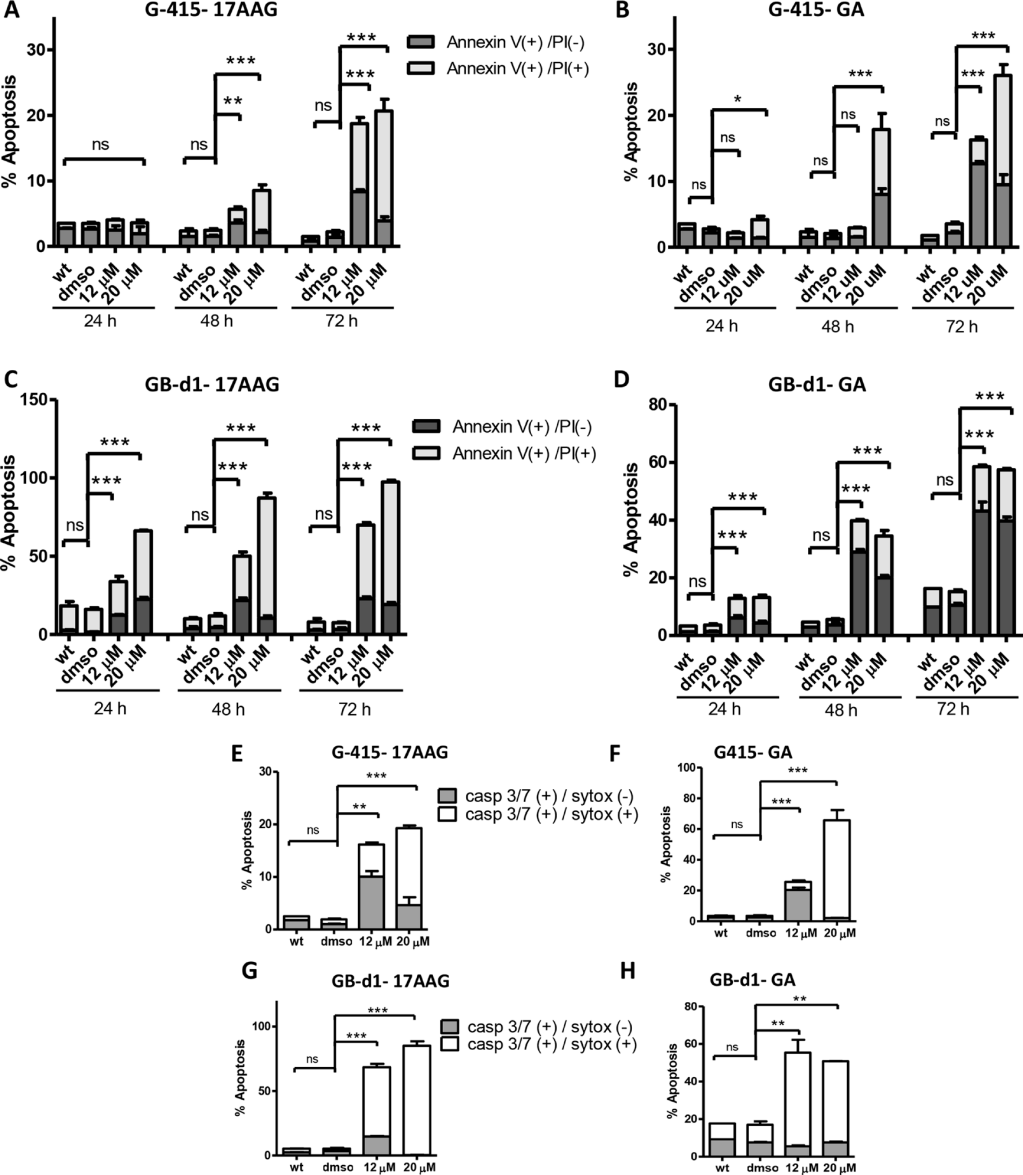
Effect of 17-AAG and GA on apoptosis in human GBC cells (**A** and **B**) G-415 and (**C** and **D**) GB-d1 cells were treated with 17-AAG or GA and harvested after 24, 48, and 72 hours for annexin V /propidium iodide (PI) staining and analized by flow cytometry. Data are shown as mean ± SD (*P < 0.05; **P < 0.01; ***P < 0.001; ns: not significant). Activity of Caspase-3/7 in G-415 cells (**E** and **F**) and in GB-d1 cells (**G** and **H**) treated with 17-AAG or GA for 72 h was assessed by Flow Cytometry using a fluorogenic substrate for detection of activated caspases 3 and 7 in apoptotic cells. Data are shown as mean ± SD (**P < 0.01; ***P < 0.001; ns: not significant).

Cell cycle arrest was followed by induction of apoptosis, as determined by FACs analysis of annexin V staining and caspase 3/7 activity. Apoptosis was evaluated after 24, 48 and 72 hours of treatment with 12 uM and 20 uM of 17-AAG and GA. For 17AAG-treated-G-415 cells, the percentage of apoptotic cells was notably higher after 72 hours of treatment, 18.7% (12 μM) and 20.7% (20 μM) compared to control cells that showed 2.2% (*P* < 0.001) (Figure 5A). Similar findings were observed after treatment with GA, where the percentage of apoptosis cells increased after 72 hours of treatment to 12.7% (12 μM) and 26.0% (20 μM) compared to control cells that showed 2.2% (*P* < 0.001) (Figure 5B). GB-d1 cells treated with 17-AAG exhibited more susceptibility to apoptosis than G-415, with 33.8% (12 μM) and 66.2% (20 μM) of apoptotic cells after 24 hours of treatment compared to untreated control (16%; *P* < 0.001). After 72 hours of treatment these cells showed 69,9% (12 μM) and 97,4% (20 μM) of apoptotic cells whereas control cells showed 7,5% of positive cells (*P* < 0.001) (Figure 5C). Using the same concentrations of GA, the amount of apoptotic cells increased to 43.2% (12 μM) and 39.7% (20 μM) after 72 hours, whereas control cells showed 10.5% (*P* < 0.001) (Figure 5D).

We confirmed these results using a CellEvent Caspase-3/7 Green Flow Cytometry Assay Kit at 72 hours after treatment. We observed that the amount of activated caspases-3 and -7 significantly increased after 17-AAG or GA treatments in both GBC cell lines. G-415 cells treated with 17-AAG showed 16.2% (12 μM) and 19.3% (20 μM) of activated caspases-3 and-7 whereas control cells showed 2,0% (*P* < 0.01 and *P* < 0.001, respectively) (Figure 5E). G-415 cells treated with GA showed 25.7%(12 μM) and 65.7% (20 μM) of activated caspases-3 and-7 whereas control cells showed 2.5% (*P* < 0.001) (Figure 5F). GB-d1 cells treated with 17-AAG showed 68.5% (12 μM) and 85.1% (20 μM) of activated caspases-3 and-7 whereas control cells showed 3.5% (*P* < 0.001) (Figure 5G). Finally, GB-d1 cells treated with GA showed 55.4% (12 μM) and 50.8% (20 μM) of activated caspases-3 and-7 whereas control cells showed 17.5% (*P* < 0.01) (Figure 5H).

### 17-AAG inhibits tumor growth on xenograft gallbladder cancer model

**Figure 6:**
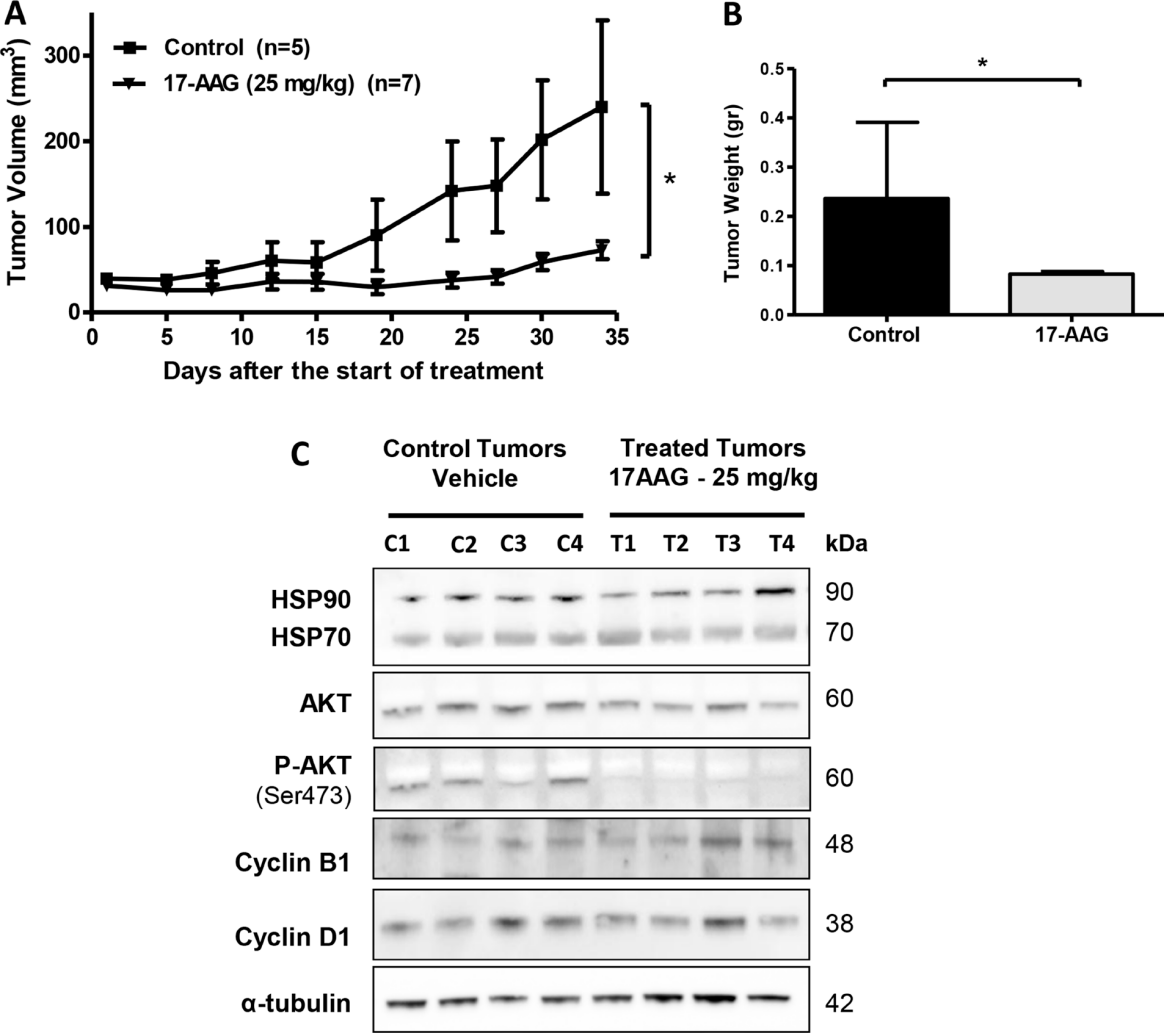
*In vivo* efficacy of 17-AAG on human GBC xenografts (**A** and **B**) Tumor growth of mice harboring G-415 tumors. G-415 cells were injected subcutaneously into NOD-SCID mice. When tumors reached a volume of approximately 50 mm^3^, they were treated with 17-AAG or vehicle, as described in the materials and methods section. Animals were sacrificed at day 34 after treatment initiation. Data are expressed as mean ± SD. 17-AAG exerted a statistically significant antitumor effect compared to the groups treated with vehicle (*P < 0.05 at day 34). (**C**) Western blot analysis of HSP90 and HSP90 clients proteins of tumor tissue from mice treated with vehicle or 17-AAG.

Based on the *in vitro* evidence, we decided to study further whether HSP90 inhibitors can be therapeutically effective on established s.c. human gallbladder tumors *in vivo*. The effect of 17-AAG on tumor growth was evaluated in xenograft GBC tumor models. Briefly, 2 × 10^6^ G-415 cells were injected subcutaneously into NOD-SCID mice. When tumors reached an average volume of 50 mm^3^, mice were treated with 17-AAG at a concentration of 25 mg/kg administered i.p., daily for 5 days per week for 4 weeks. Mice were sacrificed 34 days after initiation of the treatment and a necropsy was performed that included removal of the entire tumor area. Mice bearing G-415 tumors and treated with 17-AAG exhibited 69.6% reduction in average tumor size (*P* < 0.05), as well as 64.9% in tumor weight (*P* < 0.05) compared to the control (Figure 6A and 6B). In addition, a decrease of the Ki67 index was observed in treated mice compared to controls, although it was not statistically significant (Supplementary Figure 1). Expression of HSP90 and its target proteins in tumor tissues obtained at the end of the *in vivo* studies were assessed by western blot analysis. As shown in Figure 6C, treatment with 17-AAG inhibitor significantly decreased the expression of phospho-AKT in G-415 tumors (*p* value). However, no significant changes were observed in total AKT, HSP90, HSP70, Cyclin B1 and Cyclin D1 protein expression under the treatment conditions assayed.

### Expression levels of HSP90 in GBC tissues

**Figure 7:**
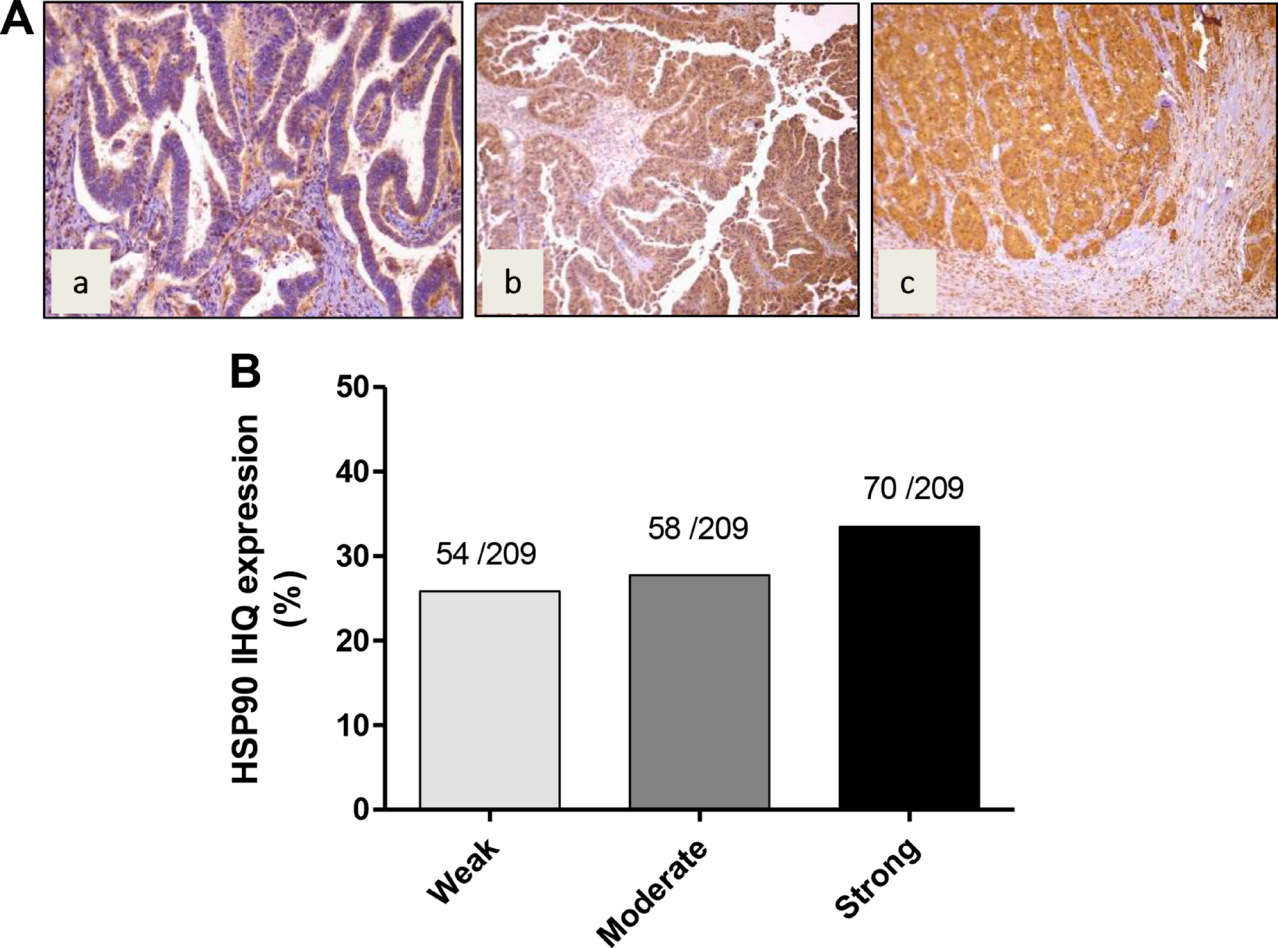
Immunohistochemical staining of HSP90 in gallbladder cancer tissue (**A**) Representative images from gallbladder lesions showing positive immunoreactivity for HSP90 in three cases of GBC: a) weak intensity b) moderate intensity c) strong intensity. (**B**) Frequency distribution of HSP90 immunohistochemical staining in gallbladder cancer.

In order to establish whether HSP90 is frequently expressed in GBC, we examined the expression of HSP90 by IHC on TMAs. Examples of staining intensity are illustrated in Figure 7, showing weak, moderate and strong staining to HSP90 in the cytoplasm of GBC cells (Figure 7A). In most cases, HSP90 showed a diffuse pattern of staining. HSP90 expression was seen in 182/209 cases of GBC (87%). HSP90 was strongly expressed in 70 cases (33%), moderately in 58 cases (28%), and weakly in 54 cases (26% ) (Figure 7B). There were no significant differences in HSP90 expression between dysplasia and early and advanced carcinoma lesions.

## DISCUSSION

High throughput rapid small molecule inhibitor screening has been successfully used to screen drugs for a series of heamatological malignancies [6]. Through the same approach we screened 130 small molecule inhibitors for GBC, which has allowed us to identify many promising compounds/drugs for using against GBC, such as, 17-AAG, Velcade, Volasertib and YM-155. As 17-AAG is a multi-target drug that acts on a diverse set of regulatory pathways [15], we decided to evaluate the therapeutic efficacy of this HSP90 inhibitor and its analog geldanamycin, *in vitro* and in preclinical models of human GBC in NOD-SCID mice. We also evaluated the expression of HSP90 by immunohistochemistry in GBC tumors.

HSP90 is a chaperone protein involved in the stabilization and conformational maturation of numerous oncoproteins [8]. Many studies have demonstrated that HSP90 is associated with the pathogenesis, poor prognosis and resistance to therapy of various human cancers [10, 11, 16, 17]. HSP90 target proteins are known to contribute to nearly every aspect of the oncogenic process including immortality, survival, anti-apoptosis, genomic instability, neoangiogenesis and metastasis [18–20]. Inhibition of HSP90 through blockade of ATP-binding sites of the HSP90-partner complex results in the dephosphorylation and/or the proteasomal degradation of these target proteins and leads to potent antitumor activity [7].

Several studies have demonstrated the therapeutic efficacy of HSP90 inhibitors on *in vitro* models and its suppression of tumor growth in numerous solid tumors in human subcutaneous growing xenografts [21, 22]. However, no studies have reported the use of HSP90 inhibitors on *in vitro* or *in vivo* GBC models. Although currently no HSP90-targeting agents have been approved for human use, several clinical trials have shown their antitumor activity, alone or in combination with other cytotoxic agents, in multiple tumor types [23]. 17-AAG (Tanespimycin) is the first HSP90 inhibitor that has been investigated in phase II clinical trials in patients with thyroid, pancreatic or kidney cancer [24], and with promising results in patients with metastatic breast cancer [25].

New water-soluble analogs of 17-AAG, such as 17-DMAG (Alvespimycin) and IPI-504 (Retaspimycin) have proved to have a longer half-life in plasma, greater oral bioavailability, superior antitumor activity in preclinical studies and lower toxicity compared with 17-AAG [12]. In the past years there has been a considerable increase in the discovery of HSP90 inhibitors, progressing from first-generation derivatives of natural products to second and third-generation fully synthetic small molecules with improved pharmacokinetic profiles [13].

Even though HSP90 is widely expressed in normal tissues, HSP90 inhibitors selectively kill cancer cells but not normal ones due to the fact that HSP90 in tumor cells is mostly in its active conformation as part of multi-chaperone complexes that have 100-fold greater binding affinity for 17-AAG compared to the uncomplexed, latent form of HSP90 that is present in normal cells [7, 26].

Currently 17-AAG is not under clinical study, but other HSP90 inhibitors, with even better pharmacological and toxicological properties, such as retaspimycin HCL, NVP-AUY922, NVP-BEP800, CNF2024/BIIB021, SNX-5422, STA-9090, are currently under investigation [27]. STA-9090 (Ganetespib), having improved pharmacologic properties in terms of potency, safety, and tolerability compared with other first- and second generation HSP90 inhibitors, has reached phase III clinical trial and currently 12 clinical trials are underway (https://clinicaltrials.gov).

The induction of cell cycle arrest and apoptosis are common mechanisms proposed for the antiproliferative effect of 17-AAG on cancer cells [28, 29]. Similar to what has been observed in other tumoral cells treated with HSP90 inhibitors [30, 31], our studies showed that 17-AAG and GA decreased cell viability and cell migration in GBC cells and these effects were strongly associated with a marked decrease of HSP90 target proteins such as EGFR, AKT, phospho-AKT, phospho-ERK, cyclin D1 and cyclin B1. Also, in concordance to other studies, we observed a marked increase in expression of HSP90 after 17-AAG or GA treatment, likely through activation of the heat shock transcription factor 1 (HSF1), the master stress-inducible regulator, in the cytoplasm and the nucleus [32–36], or by disrupting nuclear HSP90/multichaperone complexes that inhibit activation of DNA-bound HSF1 [37].

Molecular changes observed in our study were consistent with the increased G2/M cell cycle arrest and apoptosis observed in our *in vitro* studies. It is widely known that ERK1/2, Cyclin B1 and Cyclin D1 are implicated in the regulation of the G2/M phase transition and are required for mitosis to occur normally [38]. It has also been demonstrated that blockade of EGFR results in a significant inhibition of the *in vitro* and *in vivo* growth of several cell lines derived from human carcinomas of various histological types [39, 40]. In a cohort of 49 samples from patients with advanced biliary tract carcinomas (BTC), 13 of which corresponded to GBC, Pignochino *et al*., showed that EGFR was expressed in 38.5% of GBC cases, and phospho-MAPK and phospho-AKT were expressed in < 46% of these cases, indicating the activation of the EGFR pathway [40]. They also demonstrated that blocking EGFR/HER2 signaling resulted in considerable antiproliferative effects on *in vitro* models of BTC. It was also shown that targeting EGFR/HER2 pathways enhances the antiproliferative effect of gemcitabine in biliary tract and gallbladder carcinomas [40]. Inhibition of HSP90 target proteins such as AKT and phospho-AKT, which are frequently active or overexpressed in many cancer cells, including GBC, affect the PI3K/AKT/mTOR signaling pathway which plays an important role in regulating cell cycle progression, apoptosis and cell survival [41, 42].

Considering HSP90 interactions with a number of oncogenic proteins, the observed overall effect of GA and 17-AAG on the studied GBC cell lines depends on the additive effect of all these proteins [29]. This could explain that although we observed an increased expression of Cyclin B1 in G-415 cells after treatments either with GA or 17-AAG, this did not affect the inhibitory effect on proliferation and cell migration in this cell line. Interestingly, Gazitt *et al*., also reported that treatment with 17-AAG caused an upregulation of Cyclin B1 in osteosarcoma cell lines, and association of this phenomenon with mitotic blockage [43].

Our *in vivo* study demonstrated for the first time the therapeutic efficacy of a HSP90 inhibitor in GBC xenografts. The significant tumor mass reduction observed in 17-AAG-treated mice was associated with a marked decrease in phosphorylation of AKT. This is especially relevant, considering that previous studies by our group have shown upregulation of the PI3K/AKT/mTOR signaling pathway in advanced GBC [44, 45]. It is widely known that AKT protects cells from apoptosis by phosphorylating and inactivating several key apoptotic molecules [46]. Dephosphorylation and inactivation of AKT increases sensitivity of the cells to apoptosis-inducing stimulus [47]. Several studies have shown that 17-AAG sensitizes tumor cells to taxol-induced apoptosis [48] and synergizes with cisplatin inducing apoptosis in cisplatin-resistant esophageal squamous cell carcinoma cell lines [22]. 17-AAG also sensitizes radioresistant cells to radiation by inhibiting the PI3K-Akt pathway [49]. Arlander *et al*., reported that 17-AAG-mediated disruption of Chk1 activation drastically sensitized various tumor cells to gemcitabine, which is, in combination with platinum-based agents, the standard treatment for patients with advanced biliary-tract cancer [50].

Previously, our group have demonstrated that rapamycin exert strong therapeutic efficacy on subcutaneous xenograft tumors of human GBC [51]. Also, Ding *et al*., showed that the combination of rapamycin, Cl-1040 and 17-AAG reduced circulating prostate cancer cells in a mouse model for circulating prostate cancer DNA quantification [52]. These findings suggest that the combination of rapamycin and 17-AAG could be a strategy that should be considered for future studies.

Unlike *in vitro* assays, no expression changes in other proteins were observed *in vivo*. This could be explained by the dose regimen used in the treated mice, which was the dose tolerated for the animals, but clearly was not sufficient to affect most of the evaluated HSP90 target proteins from tumor samples. To optimize the *in vivo* dosing regimen and to reach a suitable efficacy-tolerability balance may require the use of other HSP90 inhibitors with even better pharmacological and toxicological properties, such as 17-DMAG, which is a water soluble analog of 17-AAG, or second or third generation HSP90 inhibitors [53, 54]. Also, more studies should be conducted to determine whether other proteins or pathways are affected by 17-AAG in GBC models.

In conclusion, our findings provide a rationale for the potential use of HSP90 inhibitors as a novel, highly effective therapeutic strategy for GBC treatment.

## MATERIALS AND METHODS

### Cell culture

Seven human GBC cell lines GB-d1, G-415, SNU308, NOZ, TGBC1TKB, TGBC2TKB and TGBC24TKB were used for the rapid small molecule inhibitor screening. These cell lines exhibit different invasive and differentiation properties. Detalis are provide in Supplementary Table 2. G-415, NOZ, TGBC1TKB, TGBC2TKB and TGBC24TKB were purchased from Riken BioResource Center (Ibaraki, Japan), GB-d1 and SNU308 were provided by Anirban Maitra (Department of Pathology, Johns Hopkins University School of Medicine, Baltimore, MD, USA). GB-d1 was authenticated by short tandem repeat analysis.

G-415 and GB-d1 were grown in Roswell Park Memorial Institute (RPMI) 1640 medium, supplemented with 10% fetal bovine serum, 2 mM glutamine, 100 U/ml penicillin and 100 μg/ml streptomycin and maintained in a 37°C atmosphere containing 5% CO_2_. The other cell lines SNU308, NOZ, TGBC1TKB, TGBC2TKB and TGBC24TKB were cultured in DMEM- high glucose with 10% fetal bovine serum, 2 mM glutamine, 100 U/ml penicillin and 100 μg/ml streptomycin and maintained in a 37°C atmosphere containing 5% CO_2_.

### Clinical samples

A total of 209 gallbladder specimens from patients who underwent curative surgical resection upon GBC diagnosis at Hospital Clinico de la Pontificia Universidad Católica (Santiago, Chile) between 1989 and 2007 were included in this study. None of the included patients with advanced GBC received any neoadjuvant or coadjuvant therapy, or radical surgery after the cholecystectomy. The clinicopathologic features of these patients were obtained from medical records and are summarized as follows: Histologically, cases were classified according to lesion type as dysplasia (*n* = 18), early carcinoma (*n* = 38), or advanced carcinoma (*n* = 171). Early and advanced carcinoma were further classified according to grade of differentiation: well-differentiated (*n* = 22), moderately differentiated (*n* = 81), and poorly differentiated (*n* = 106).

### HSP90 inhibitors

17-Demethoxy-17-allylaminogeldanamycin (17-AAG) inhibitor (A6880) was purchased from LC Laboratories (Woburn, MA, USA) and geldanamycin (GA) (ant-agl-5) was purchased from InvivoGen (San Diego, CA, USA). For *in vitro* assays the inhibitors were resuspended to 10 mM in dimethylsulfoxide (DMSO) as stock solutions and stored at −20°C. Inhibitors were diluted in culture medium before each *in vitro* experiment and 0.01% DMSO in culture medium was used as a vehicle control. For *in vivo* studies 17-AAG was dissolved in DMSO at 25 mg/ml and stored at −20°C.

### Antibodies

Mouse monoclonal antibody anti-HSP90 (NCL-HSP90) was purchased from Novocastra Laboratories (Newcastle-upon-Tyne, UK) and used for both western blot and immunohistochemical analyses. Mouse monoclonal antibody anti-EGFR (#280005) was purchased from Invitrogen Life Technologies (Grand Island, NY, USA), anti-Cyclin B1 (SC-245) was purchased from Santa Cruz Biotechnology (Santa Cruz, CA, USA). Rabbit monoclonal antibodies anti-AKT, clone 11E7 (#4685), anti-phospho-AKT, clone D9E (Ser473, #4060), anti-p44/42 MAPK (ERK1/2) (#4695), anti-phospho-p44/42 MAPK (Erk1/2) (Thr202/Tyr204, #4370), anti-survivin (#2808) were purchased from Cell Signaling Technology (Danvers, MA, USA), anti-α-tubulin (32–2500) was purchased from Invitrogen Life Technologies and Cyclin D1, clone EP12 (M364201) was purchased from Dako North America Inc. (Carpinteria, CA, USA) and used for Western blot analysis. The secondary antibodies anti-rabbit IgG, goat HRP-linked (#7074) and anti-mouse IgG, goat HRP-linked (#7076) were purchased from Cell Signaling Technology. For Ki67 index, automated staining was performed using an antibody against Ki67 antigen (Clone MIB1 DAKO) at a dilution of 1:100. Ki67 index was measured using the open access web application ImmunoRatio [55] for automated image analysis using 4–10 images per sample, depending on tumor size and excluding necrotic areas. Statistical analyses were performed using an unpaired Mann Whitney test using GraphPad Prism v. 6.

### Small molecule inhibitor screening on gallbladder cancer cell lines

Cells were seeded into 384 well assay plates at 400 cells/well in their respective media. Inhibitors were obtained from LC Laboratories (Woburn, MA, USA) and Selleck Chemicals (Houston, TX, USA) were dissolved in DMSO and added at a concentration series ranging from 10 mM to 0.01 mM as previously described [5]. The final concentration of DMSO used was 0.01% throughout the screening methods. After three days of culture at 37°C, 5% CO_2_, CellTiter96 (Promega Madison, WI, USA) was added and optical density was measured at 490 nm and used to determine cell viability. The concentration of inhibitor required to inhibit cell growth by 50% (IC_50_) was calculated using a non-linear regression analysis.

### Cell viability assay

G-415 and GB-d1 cell lines were plated into 96 well plates at a density of 3 x 10^3^ cells per well. After an overnight attachment period cells were treated either with 17-AAG, GA or 0.01% dimethylsulfoxide (as control). The number of viable cells was determined at 24, 48, and 72 hours using CellTiter 96 Aqueous One Solution Cell Proliferation assay (Promega Corp., Madison, WI). Briefly, 20 μl CellTiter 96 solution was added to each well and the plates were incubated for 1 hour after which the absorbance of each well was read at a wavelength of 490 nm using a multiwell plate reader (Autobio Labtec Instruments, Zhengzhou City, China). All assays were performed in five technical replicates, and each assay was repeated three times.

### Transwell cell migration assay

Migration assays were performed using 24-well Transwell™ plates containing polycarbonate filters with an 8 μm pore size (BD Biosciences, Bedford, MA, USA). Complete medium was placed in the lower chamber to act as a chemoattractant and 5 × 10^4^ cells of either G-415 or GB-d1 cells were seeded with serum-free medium into the upper chamber. Cells were exposed to 17-AAG (12 μM), GA (15 μM) or 0.01% DMSO (as control) for 24 hours at 37°C. After 24 hours, cells were fixed in methanol for 15 minutes and stained with 0.05% crystal violet in 25% methanol/PBS for 15 minutes. Cells on top of the membrane were removed using a cotton swab, and filters were washed with PBS. Cells on the underside of filters were viewed and counted under a microscope in 10 randomly selected fields.

### Western blot analysis

For *in vitro* assays, cells were seeded on 75 cm^2^ culture plates. Cells at 60–70% were treated with 17-AAG (12 μM), GA (15 μM), or 0.01% dimethylsulfoxide (as control) for 24 hours. Cell lysates were prepared using RIPA buffer (Sigma Aldrich, St Louis, MO, USA). Briefly, cells were washed three times with cold PBS and lysed on ice with RIPA buffer containing a phenylmethylsulfonyl fluoride (PMSF), protease and phosphatase inhibitor cocktail (Sigma Aldrich, St Louis, MO, USA). For *in vivo* experiments, tumor lysates were prepared on ice using cell lysis buffer (1% Triton X-100, 150 mM NaCl, 50 mM Tris-HCl pH 7.4, 2 mM EDTA) containing a protease and phosphatase inhibitor cocktail (Sigma Aldrich, St Louis, MO, USA). Whole lysates were collected after centrifugation at 14.000 rpm for 10 minutes at 4°C. Protein concentrations were determined using a BCA Protein Assay Kit (Pierce, Thermo Fisher Scientific Inc, Rockford, IL, USA) according to the manufacturer's instructions. Equal amounts of total cellular protein (40 μg) were separated by sodium dodecyl sulfate-polyacrylamide gel electrophoresis in 4%–20% Mini-PROTEAN TGX Gels (Bio-Rad Laboratories Inc, Hercules, CA, USA) and electrotransferred to PVDF membranes (Immobilon^®^-P membrane Millipore, Bedford, MA, USA). Membranes were blocked with 1X Tris-buffered saline containing 0.05% Tween (TBST) and 5% fat-free milk for 1 hour at room temperature and incubated overnight at 4°C with primary antibodies. After washing with TBST, membranes were further incubated with the secondary antibodies for 1 hour at room temperature. Antibody-bound protein bands were detected using SuperSignal™ West kit (Pierce, Thermo Scientific Inc, Rockford, IL, USA). Images were acquired with the ImageQuant LAS 500 system (GE Healthcare Life Sciences, Pittsburgh, PA, USA ). Alpha-tubulin expression was used as a loading control.

### Cell cycle analysis

G-415 and GB-d1 cell lines were seeded into 6 well plates at a density of 1x10^5^ cells per well. After overnight incubation, cells were treated either with 17-AAG (12 μM), GA (15 μM) or 0.01% dimethylsulfoxide (as control) for 24 hours. Cells were trypsinized, washed and fixed in 70% ethanol at −20°C overnight. Cells were then washed and incubated for 30 minutes at room temperature and in darkness in a solution containing 20 μg/ml Propidium Iodide (PI) (Sigma Aldrich, St. Louis, MO, USA), 100 μg/ml RNase (US Biological, Salem, MA) and 0,1% Triton X-100). Cells were evaluated on a FACSCanto II (Becton Dickinson, Franklin Lake, NJ) and data was analyzed with BD FACSDiva software, version 6.0. Experiments were performed in three technical replicates, and data was expressed as mean ± S.D.

### Apoptosis analysis

G-415 and GB-d1 cell lines were plated into 6 well plates at a density of 1 × 10^5^ and 6 × 10^4^cells per well, respectively. After an overnight attachment period cells were treated either with 17-AAG, GA or 0.01% dimethylsulfoxide for 24, 48 and 72 hours. Cells were harvested by trypsinization and apoptosis was determined using two different methods, Alexa Fluor 488 annexin V/Dead Cell Apoptosis Kit (Invitrogen, Eugene, OR) and CellEvent Caspase-3/7 Green Flow Cytometry Assay Kit (Life Technology, Carlsbad, CA), according to the manufacturer's protocol. Data were collected and analysed on FACSCanto II (Becton Dickinson, Franklin Lake, NJ) using BD FACSDiva software, version 6.0. Annexin V (+)/PI (−) and annexin V (+)/PI (+) cells were considered early and late apoptotic cells, respectively, and both were counted as total apoptotic cells. Caspase 3/7 (+)/Sytox (−) and caspase 3/7 (+)/Sytox (+) also were counted and considered as total apoptotic cells. Experiments were performed in three technical replicates and a total of 10,000 cells were analyzed in each individual experiment.

### *In vivo* tumor xenograft assays

8 to 12-week-old NOD-SCID mice (obtained from Jackson Laboratory, Bar Harbor, USA) were subcutaneously injected in one flank with 2 × 10^6^ cells of G-415 resuspended in 200 μl of PBS with 30% of Matrigel (Matrigel™ Basement Membrane Matrix, BD Biosciences). When the average tumor reached 50 mm^3^, mice were randomly separated into two groups and treated with 17-AAG and its respective vehicle. 17-AAG was administered at a daily intraperitoneal (i.p) dose of 25 mg/kg for 5 days per week for 4 weeks. Tumor volumes were estimated twice a week from caliper measurements (volume = 0.52 × (width)^2^ × length).

### HSP90 immunohistochemical staining

Whole tissue sections (WTSs) and tissue microarrays (TMAs) were used for this study. Three different representatives areas were sampled for TMAs construction according to previously published methods [56]. None of these patients had received preoperative chemotherapy. HSP90 expression was evaluated using a previously described immunohistochemistry protocol [56]. Briefly, heat induced antigen retrieval was performed prior to immunohistochemical staining. Sections were incubated with a HSP90 monoclonal antibody (1:200 dilution, Novocastra Laboratories) at 4°C overnight. Detection of signal was achieved using the LSAB + kit (DAKO, Carpinteria, CA, USA) according to the manufacturer's recommendations. 3,3′-diaminobenzidine/H_2_O_2_ (DAKO) was used as chromogen and slides were counterstained with hematoxylin. We used gastric cancer tissue as positive control and omission of the primary antibody was used as a negative control. The immunohistochemical staining of HSP90 was examined by two independent and specialized pathologists (JRV, AHC) without any information about clinicopathological features or prognosis. Immunohistochemical staining was evaluated based on staining intensity and positive cells within the whole tissue section using a previously described scoring system [56]. Briefly, an arbitrary 20% cutoff was used to determine HSP90 positivity. Only cytoplasmic staining was considered positive for HSP90, and expression was graded visually as negative, weak or moderate/strong [56].

### Ethics statement

Use of gallbladder specimens from patients was approved by the IRB of the School of Medicine, Pontificia Universidad Católica de Chile. Mouse husbandry and animal experiments has been conducted in accordance with the ethical standards and according to the local animal protection law.

### Statistical analysis

Statistical analyses of *in vitro* and *in vivo* experiments were performed by analysis of variance (one way ANOVA) followed by Tukey's multiple comparisons test. *P* values < 0.05 were considered significant. Data analysis was performed with the GraphPad Prism 5 (GraphPad Software, Inc. San Diego CA).

## SUPPLEMENTARY MATERIALS FIGURES AND TABLES


