# Supplementary material for: Small molecule inhibitor screening identifified HSP90 inhibitor 17-AAG as potential therapeutic agent for gallbladder cancer
Source: Oncotarget. 2017 Feb 16;8(16):26169–84. doi: 10.18632/oncotarget.15410 (PMC5432248; doi:10.18632/oncotarget.15410)
Supplement: Supplementary file 1 [file oncotarget-08-26169-s001.pdf]

Small molecule inhibitor screening identifified HSP90 inhibitor 17-AAG as potential therapeutic agent for gallbladder cancer

Supplementary Materials

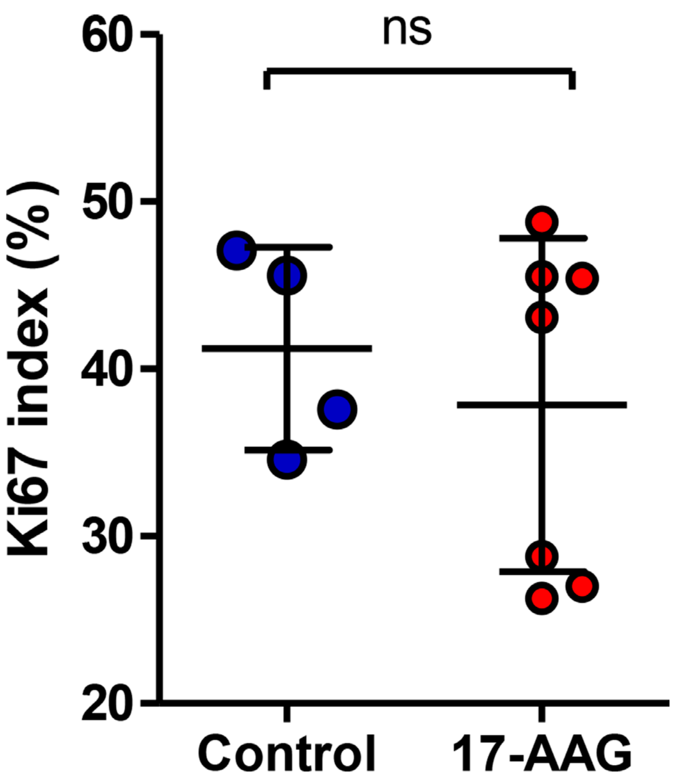

Supplementary Figure 1: Ki67 Immunostaining of GBC xenografts. Ki67 proliferation index was assessed by immunohistochemistry in G-415 xenografts collected 34 days after starting the treatment. Ki67 positive cells were counted to calculate the Ki67 proliferation index. Data are expressed as mean ± SD. (ns: not significant).

Supplementary Table 1: List of drugs and IC50 (nM). See Supplementary_Table_1

Supplementary Table 2: Gallbladder cancer cell lines used in the rapid small molecule inhibitors

Cell line	*In vitro* Invasion Phenotype	Case history	Tissue derived from	Gender	Age	Differentiation
GB-D1	High	Adenocarcinoma	Gallbladder	M	58	Poor
G-415	High	Carcinoma	Gallbladder	M	68	Undifferentiated
SNU308	Less	Adenocarcinoma	Gallbladder	NA	NA	Moderate
NOZ	Moderate	Adenocarcinoma	Gallbladder	F	48	Moderate
TGBC1TKB (M)	Less	Carcinoma	Gallbladder, lymph node	F	NA	NA
TGBC2TKB (P)	Less	Carcinoma	Gallbladder	F	NA	NA
TGBC24TKB	Non invasive	Carcinoma	Gallbladder, ascites	F	NA	NA
